# Supplementary material for: Plant sulphur metabolism is stimulated by photorespiration
Source: Commun Biol. 2019 Oct 16;2:379. doi: 10.1038/s42003-019-0616-y (PMC6795801; doi:10.1038/s42003-019-0616-y)
Supplement: Supplementary file 1 — Supplementary Information [file 42003_2019_616_MOESM1_ESM.pdf]

Supplementary Figures:

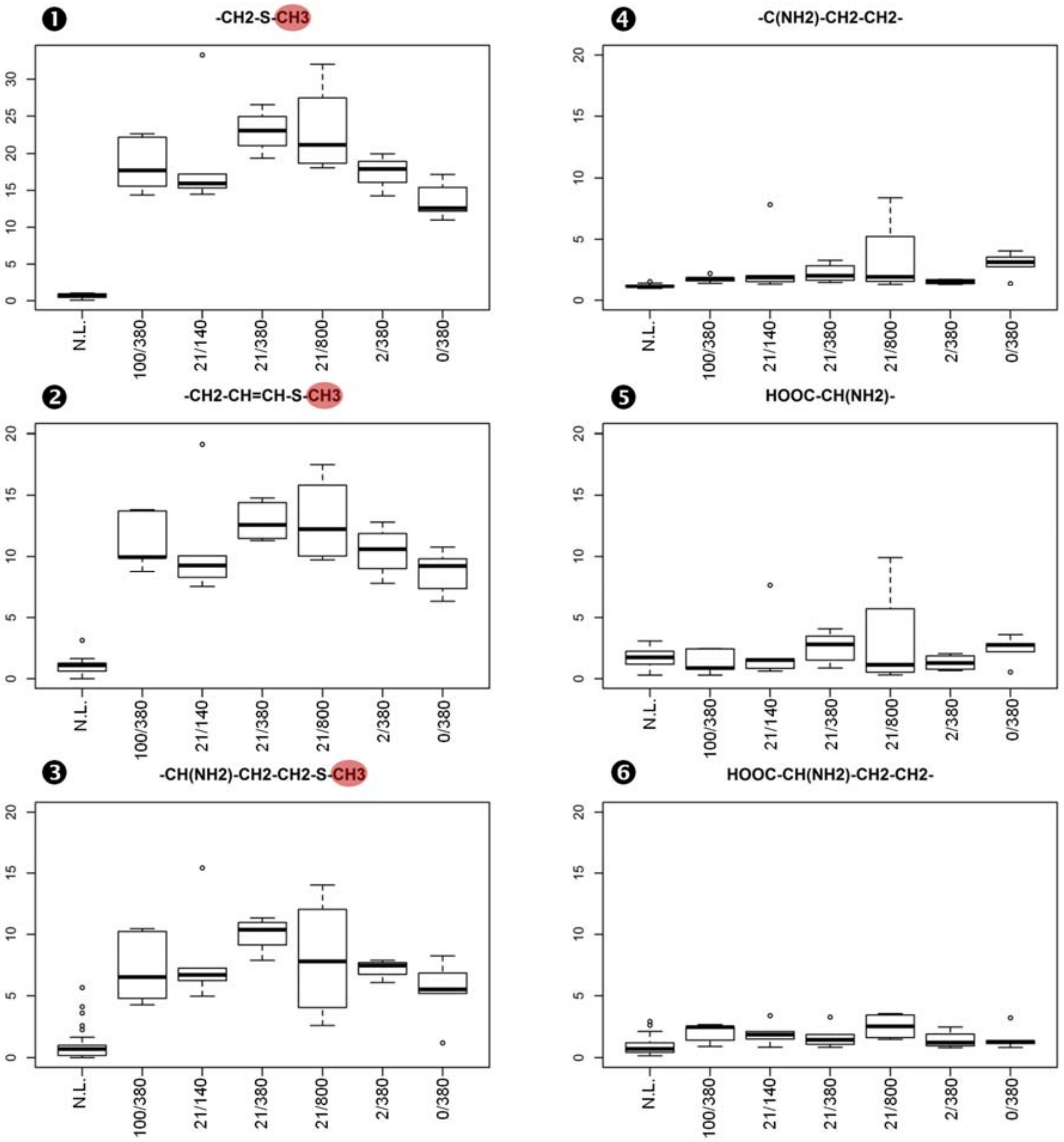

Supplementary Figure 1. Isotopic enrichment in methionine fragments observed by LC-MS in sunflower leaves labelled with ^{33}S-sulphate and $^{13}CO_2$ in the light under different O_2/CO_2 conditions. The isotopic enrichment in indicated in %^{13}C using whisker plots (median, 1st and 3rd quartiles, minimum and maximum). Conditions (O_2 in %, CO_2 in µmol mol^{-1}) are shown on the *x* axis from high to low photorespiration (N.L.: no labelling). Fragments that contain the methyl group are on the left (methyl group highlighted in red). Numbers refer to fragment numbers in Supplementary Notes. Data are from *n* = 7 biological replicates for all conditions.

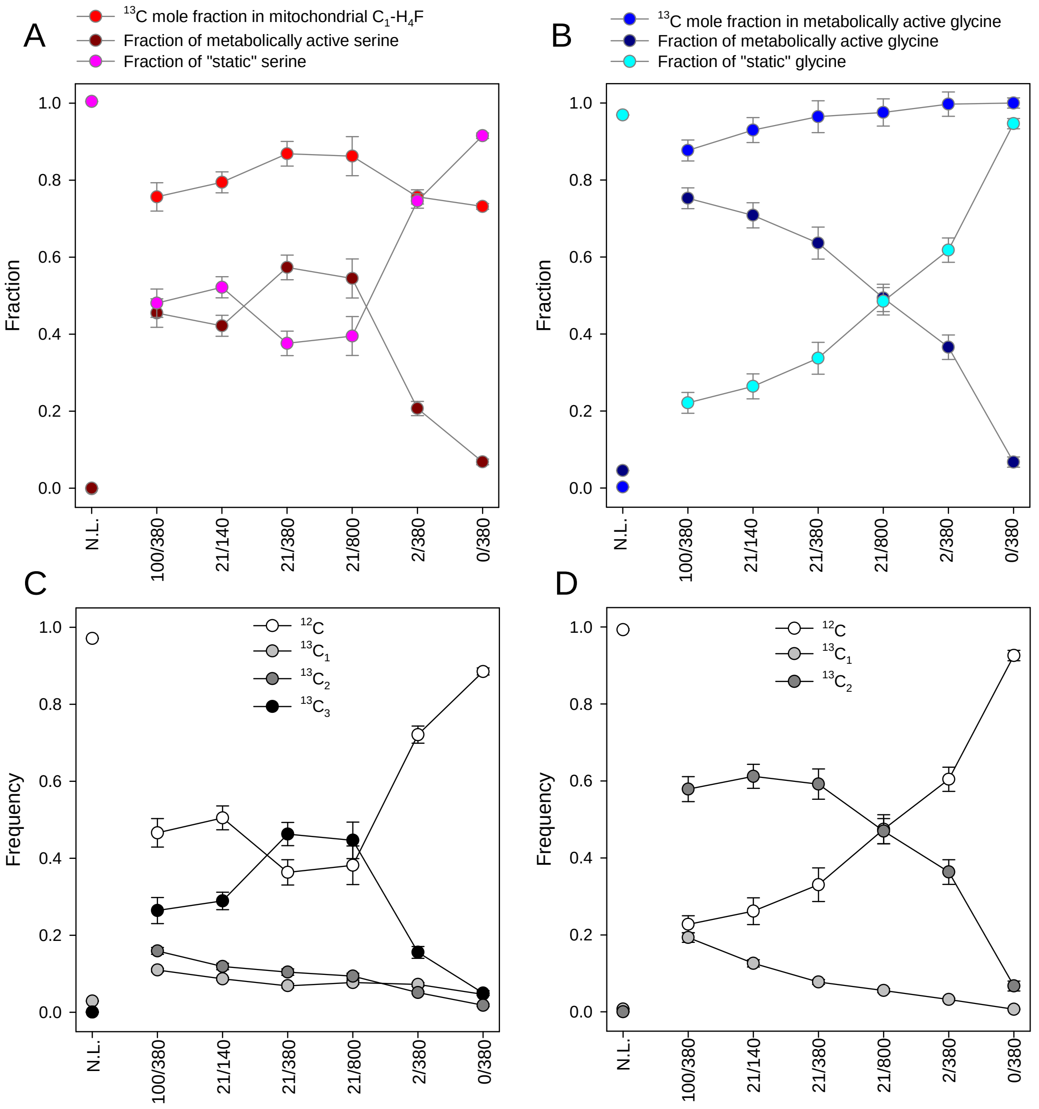

Supplementary Figure 2. Isotopic pattern in serine and glycine in sunflower leaves labelled with ^{33}S-sulphate and $^{13}CO_2$ in the light under different O_2/CO_2 conditions: **A,** calculated fraction of active and inactive (static) serine pool, ^{13}C fraction in C_1 units carried by H_4F in the mitochondrion. **B,** fraction of active and static glycine pools, and ^{13}C mole fraction in C-atoms of metabolically active glycine. **C**, serine isotopomer frequencies. **D,** glycine isotopomer frequencies. Conditions (O_2 in %, CO_2 in µmol mol^{-1}) are shown on the *x* axis from high to low photorespiration. The non-labelled situation (N.L.) is also shown as a control. Details on calculations are provided in Supplementary Notes. Mean±SE, *n* = 7 biological replicates.

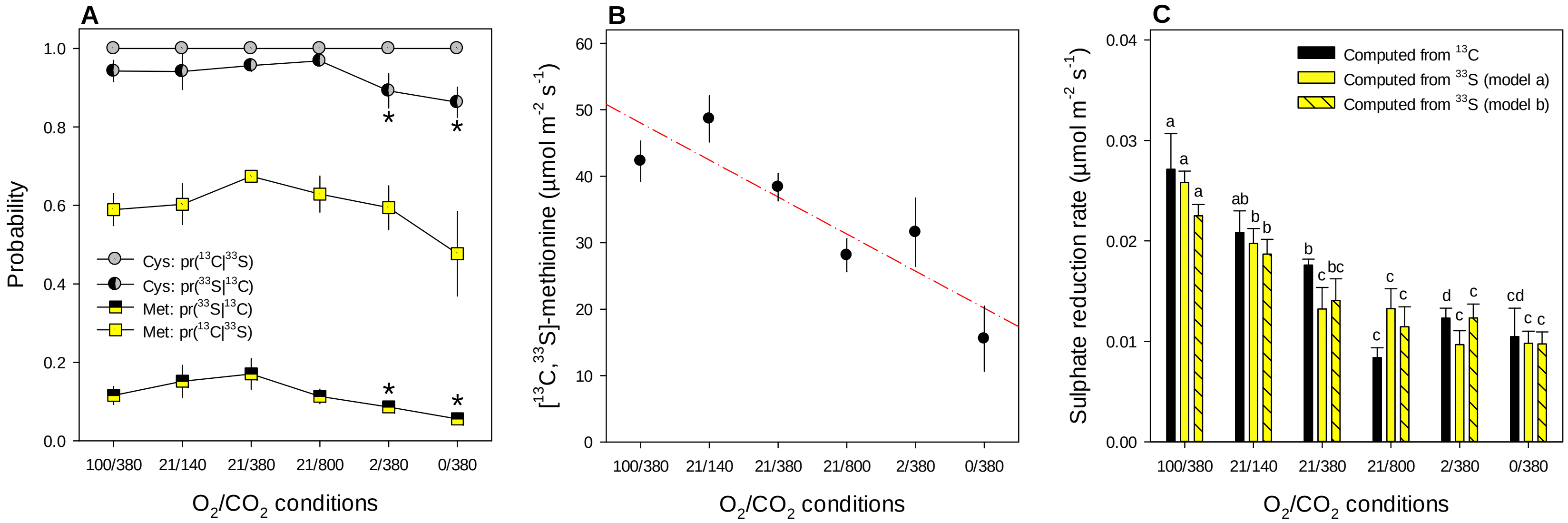

Supplementary Figure 3. Outputs of calculations from isotopic patterns: **A,** Bayes probability of finding ^{13}C-labelled molecules (regardless of the C-atom position) amongst ^{33}S-molecules (open symbols) and conversely, of finding ^{33}S-labelled molecules amongst ^{13}C-molecules (half-closed symbols) in methionine (yellow) and cysteine (grey). Note the much higher probability to find a ^{13}C in ^{33}S-molecules than the reverse in methionine (yellow symbols). **B**, calculated amount of doubly labelled methionine, i.e. with both ^{33}S and ^{13}C at the C-atom position C-5 (methyl); **C,** sulphate reduction rate calculated from ^{13}C data (black) or ^{33}S data (yellow), with model a (build-up) or b (steady-state). In B, the dotted line represents a linear regression when conditions are converted to numeric values from 1 to 6 (which is significant; $R^2 = 0.79$). In C, letters stand for statistical classes when comparing conditions for each computation ($P<0.05$). The black asterisks stand for statistical significance when compared to 'standard' ambient conditions 21/380 ($P<0.05$). Models used to compute the sulphate reduction rate and calculations of doubly labelled methionine are described in Supplementary Notes. Conditions (O_2 in %, CO_2 in µmol mol^{-1}) are shown on the *x* axis from high to low photorespiration. Mean±SE, $n = 7$ biological replicates.

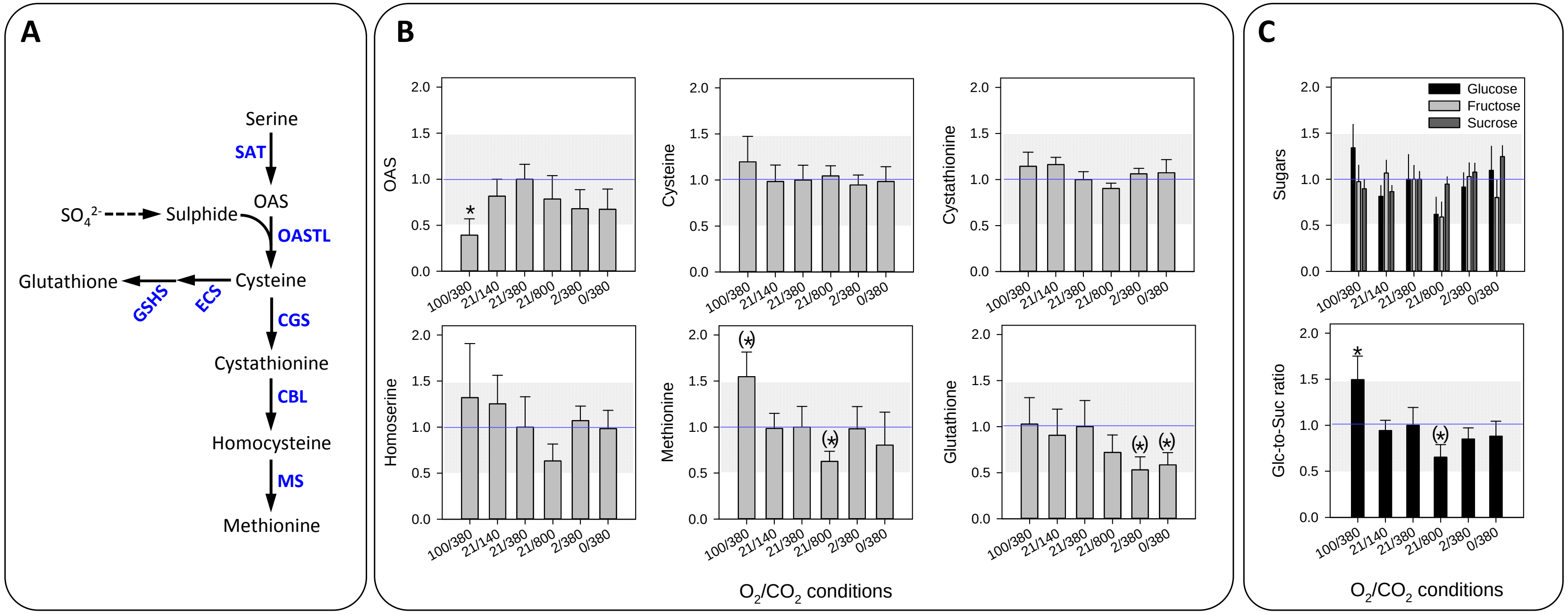

Supplementary Figure 4. Metabolite contents in illuminated leaves under different O_2/CO_2 conditions. **A**, summary of the metabolic pathway associated with S assimilation. **B**, relative quantity of S assimilation intermediates from GC-MS profiling. **C**, relative content in sugars: glucose, fructose and sucrose (top) and glucose-to-sucrose ratio (bottom). In **B** and **C**, quantities are expressed relative to the content under 'standard' conditions (21% O_2, 380 µmol mol^{-1} CO_2) fixed at 1 (blue line). The shaded area represents the ±50% region. Asterisks show a significant difference with 'standard' conditions ($P<0.05$); parenthesed asterisks stand for near-significance ($P<0.07$). Data shown are mean±SE (n = 7 biological replicates). Abbreviations: CBL, cystathionine β-lyase; CGS, cystathionine γ-synthase; ECS, γ-glutamylcysteine synthetase; GSHS, glutathione synthetase; MS, methionine synthase; OAS, *O*-acetyl-serine; OASTL, OAS thiol-lyase; SAT, serine acetyltransferase.

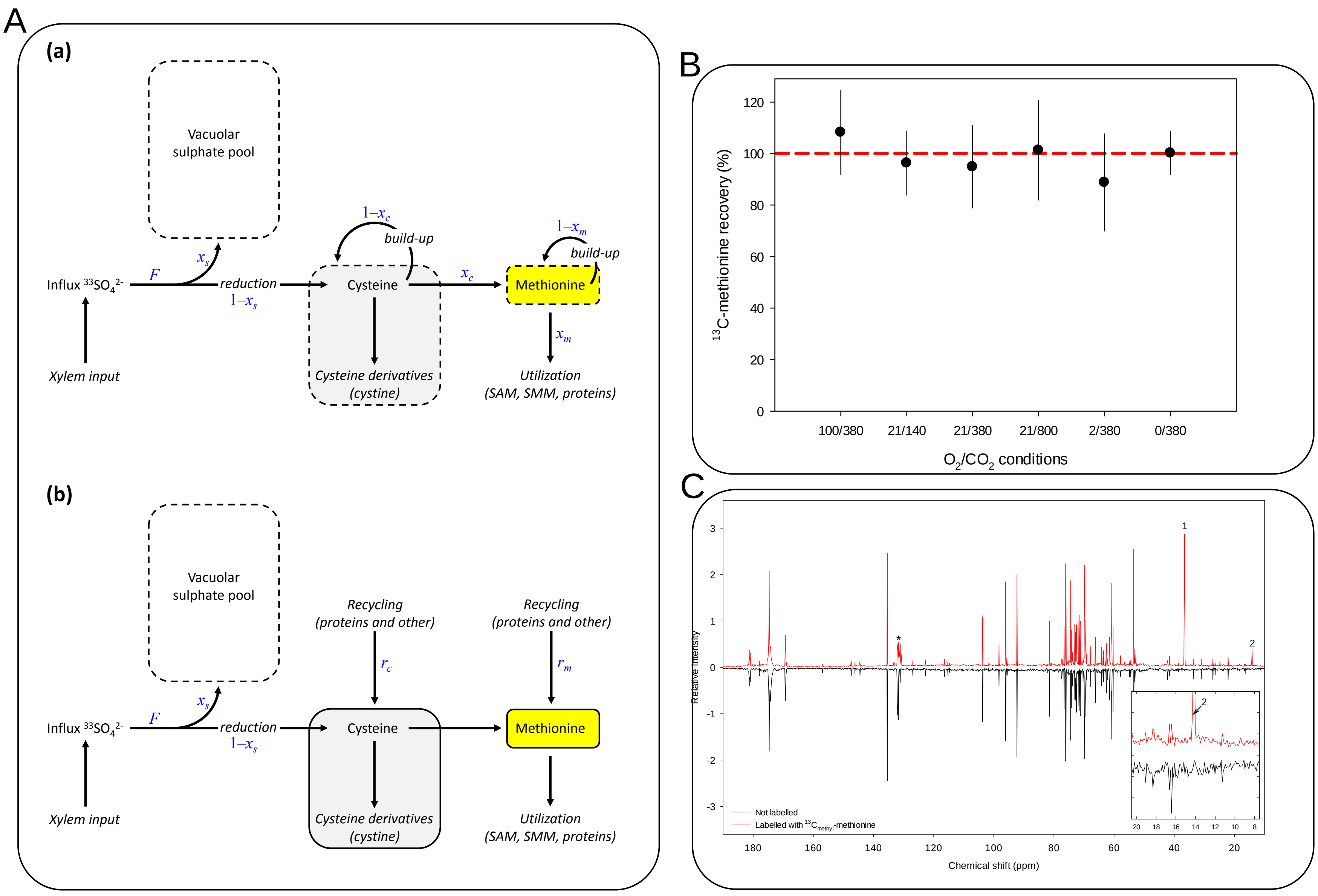
A
(a)
Vacuolar sulphate pool
Influx $^{33}SO_4^{2-}$
F
x_s
reduction
$1-x_s$
Xylem input
Cysteine
$1-x_c$
build-up
Cysteine derivatives (cystine)
x_c
Methionine
$1-x_m$
build-up
x_m
Utilization (SAM, SMM, proteins)
(b)
Vacuolar sulphate pool
Influx $^{33}SO_4^{2-}$
F
x_s
reduction
$1-x_s$
Xylem input
Recycling (proteins and other)
r_c
Cysteine
Cysteine derivatives (cystine)
Recycling (proteins and other)
r_m
Methionine
Utilization (SAM, SMM, proteins)
B
^{13}C-methionine recovery (%)
0
20
40
60
80
100
120
100/380
21/140
21/380
21/800
2/380
0/380
O_2/CO_2 conditions
C
Relative intensity
3
2
1
0
-1
-2
-3
1
2
*
Not labelled
Labelled with $^{13}C_{methyl}$-methionine
20 18 16 14 12 10 8
180
160
140
120
100
80
60
40
20
Chemical shift (ppm)

Supplementary Figure 5. Models to compute the S assimilation flux from ^{33}S data and experimental verification of related assumptions: A, the two models of ^{33}S redistribution used to compute the sulphate reduction rate from isotopic data in sulphate, methionine and cysteine. In model (a), the cysteine (+ derivatives such as cystine) and methionine pools are not in the steady-state and can vary (possible build-up). In model (b), pool sizes are fixed but cysteine and methionine can be regenerated by recycling of non-labelled compounds (such as protein turn-over) thereby leading to an isotopic dilution. In both models, partitioning coefficients are denoted as x. For example, the flux of ^{33}S-sulphate build-up is $F{\cdot}x_s$ while the flux of sulphate reduction is $F{\cdot}(1\text{–}x_s)$. SAM, S-adenosyl methionine; SMM, S-methyl methionine. The model is solved for x and r values using both ^{33}S pools (measured by NMR) and %^{33}S (measured by LC-MS). F represents the ^{33}S-influx by ^{33}S-suplhate feeding to the leaf. The vacuolar sulphate pool is not only made of 33S-sulphate from feeding but also ^{33}S-sulphate from stored vacuolar sulphate at natural abundance that could be measured by NMR as well in non-labelled leaves (about 20 µmol ^{33}S m^{-2}). In both models, it is assumed that fed ^{33}S-sulphate can be used directly by reduction and does not first incorporate the vacuolar sulphate pool, so as to reflect the fact that when fed to the leaf, sulphate arrives in the cytoplasm of mesophyll cells before being allocated to the vacuolar store, and that the isotopic enrichment in cysteine is much higher than that of sulphate (Fig. 1). In both models, cysteine and its derivatives as considered as single pool simply because with our protocol, cysteine, cysteine and glutathione have the same chemical shift (cysteinyl SO_3^- group) and thus the ^{33}S-NMR signal obtained by NMR integrates all of them. This is in contrast with methionine, because derivatives such as SMM and SAM are not visible by NMR (in practice, their concentration is too small and their peak would be too large to be detectable by ^{33}S-NMR). However, in sunflower, methionine derivatives are quantitatively minor compared to methionine (panels B and C). Note that in model (a), x_c and x_m can be, at first glance, viewed as proportions but they can be larger than 1. In that case, it simply means that pool sizes decline. By contrast, x_s is a proportion representing the partitioning of the influx of fed sulphate (F) to accumulation (x_s) and utilization ($1\text{–}x_s$), and thus it is < 1. In model (b), recycling fluxes r_c and r_m can have any positive value. In both models, the possible utilization of cysteine by other pathways such as protein synthesis is neglected. In fact, protein synthesis represents a carbon flux of at most 0.1 µmol C m^{-2} s^{-1}, meaning a sulphur flux of less than 0.002 µmol S m^{-2} s^{-1} (protein S:C ratio of at most 1:50), which is small compared to the sulphur assimilation rate. **B, recovery of ^{13}C-methionine in sunflower leaves** labelled with 15 mM ^{13}C-5-methionine (^{13}C on the methyl group) via the transpiration stream. The recovery is calculated as the amount (in moles) of ^{13}C-methionine found in the NMR spectrum of the leaf extract relative to the amount of ^{13}C absorbed by the leaf via the transpiration stream (obtained from the transpiration rate). Regardless of gaseous conditions used during illumination (shown in O_2/CO_2, in %/ppm), the recovery is very close to 100%, showing negligible utilization of methionine by protein synthesis under our experimental conditions. Mean±SD, n = 3 biological replicates. **C, ^{13}C-NMR spectrum of sunflower leaves fed with ^{13}C-methionine** (positionally ^{13}C labelled on the methyl group; red) or methionine at natural abundance (black) via the transpiration stream, in the light (21%, 380 µmol mol^{-1} CO_2). Spectra are mirrored to facilitate comparisons. Each spectrum is the average of 7 spectra. Note that the preparation of the NMR perchloric extract oxidizes about 90% of leaf methionine to methionine sulfoxide. 1, methyl group of methionine sulfoxide; 2, methyl group of (non-oxidized) methionine. Asterisk, C-2 and C-3 atoms of the internal standard (maleate), the chemical shift of which changes slightly between samples due to slight differences in pH. Note that apart from methionine and methionine sulfoxide methyl groups, there is no other ^{13}C difference between the spectra, showing negligible metabolic redistribution of added methionine.

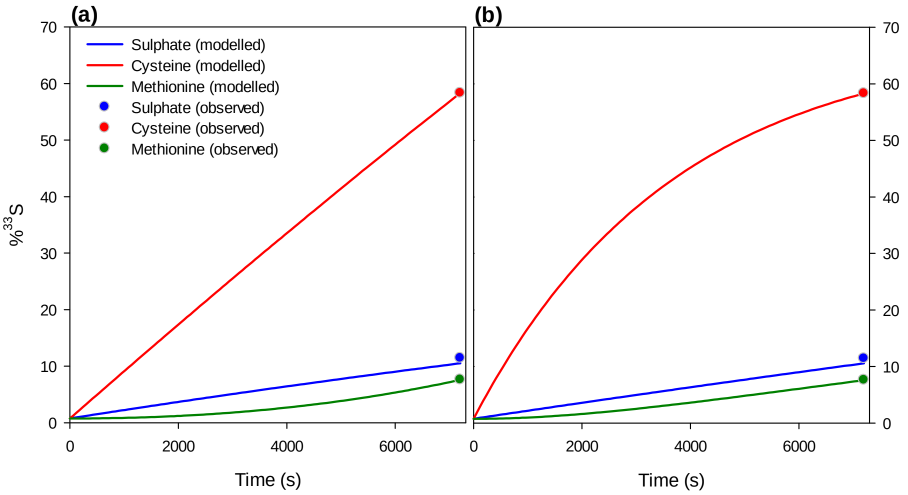

Supplementary Figure 6. Example of model output: model (a) (left) and model (b) (right). In both panels, observed values are indicated with filled discs. t = 0 represents the onset of ^{33}S-labelling. The difference in kinetics is small between models, except for cysteine, the ^{33}S-percentage of which increases nearly linearly in the variable pool size model (model a). Model outputs are extremely close, with a computed sulphate assimilation rate of 23.1 nmol m^{-2} s^{-1} (model a) and 22.5 nmol m^{-2} s^{-1} (model b). Models (a) and (b) are illustrated in Supplementary Figure 5A.

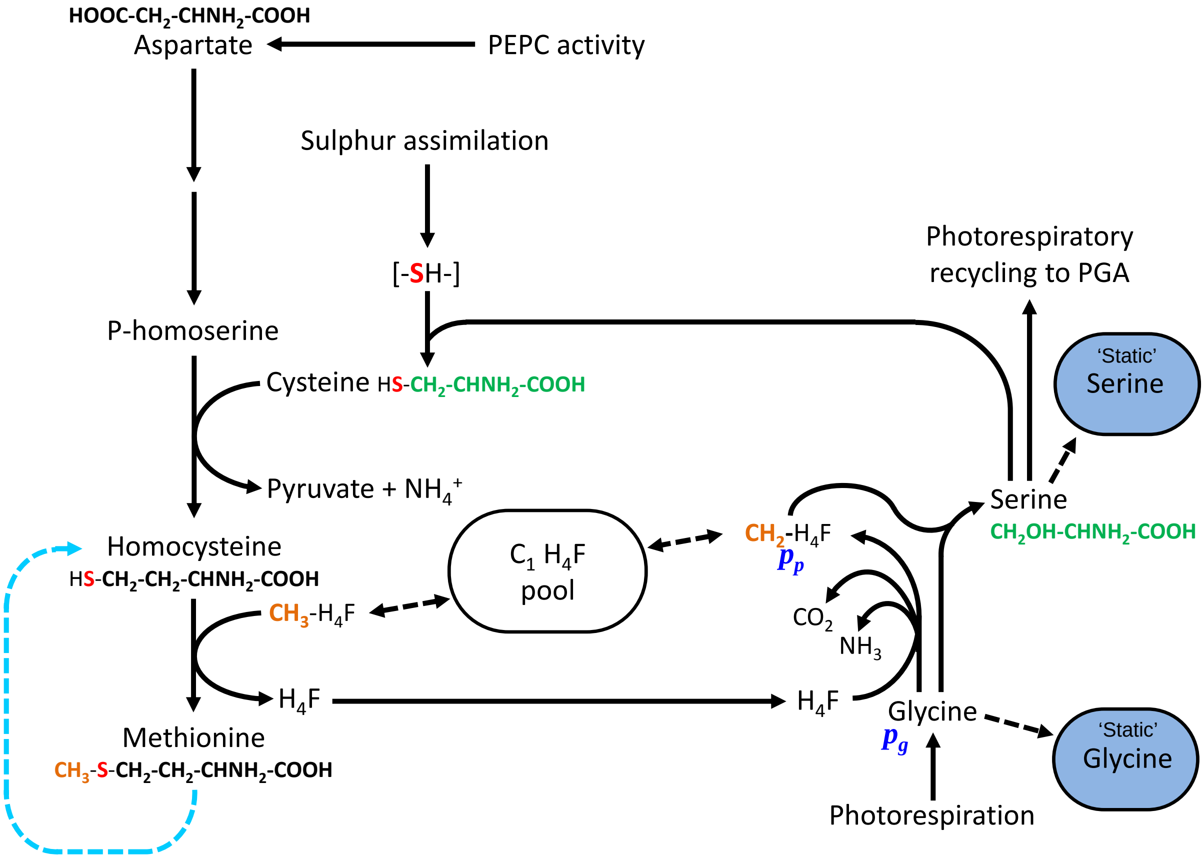

Supplementary Figure 7. Simplified metabolic pathway showing the origin of atoms in methionine, with sulphur coming from cysteine and carbon atoms coming from aspartate (C-1 to C-4, black) and methyl tetrahydrofolate (C-5, orange). De novo synthesis of cysteine utilizes serine (green) which is ^{13}C-labelled by $^{13}CO_2$ via photorespiration. The CH_3 group used to synthesize methionine can also be ^{13}C-labelled via photorespiration. Glycine and serine have both a metabolically "static" (slowly turned-over; blue bubbles) and a metabolically "active" (rapidly turned-over) pool. Tetrahydrofolate (H_4F) metabolism is subdivided in pools that are all metabolically active: methionine is synthesized in the chloroplast while the glycine-to-serine conversion takes place in the mitochondrion. However, the metabolically active glycine pool is rapidly ^{13}C-labeled and therefore so is CH_3-H_4F coming from glycine decarboxylation. Methionine can be used via S-adenosylmethionine as a CH_3 donor and this can regenerate homocysteine via the S-adenosylhomocysteine cleavage or the S-methylmethionine cycle (turquoise dashed arrow). Therefore, the methyl group in methionine is turned-over more rapidly than the S atom and thus the ^{13}C-enrichment in the methyl group can be anticipated to be larger than the ^{33}S-enrichment upon double labelling. In other words, the probability of finding a ^{13}C amongst ^{33}S-labelled methionine molecules must be higher than the probability of finding a ^{33}S amongst ^{13}C-labelled methionine molecules.

Supplementary Notes:

Supplementary Notes 1. Calculation of ^{33}S and ^{13}C enrichments from LC-MS data

High resolution LC-MS data provide the quantity of each of isotopomer/isotopologue i (denoted as x_i) amongst the I isotopic forms of the molecule of interest (monoisotopic, isotopically substituted once, twice, etc.). The overall isotopic enrichment in ^{13}C (or ^{33}S) can be simply calculated as:

$$p_{\text{overall}} = \frac{\sum_{i=0}^{I} x_i n_i}{N \sum_{i=0}^{I} x_i}$$

Where N is the total number of C (or S) atoms and n_i is the number of ^{13}C (or ^{33}S) in isotopomer (isotopologue) i. Here, the subscript "overall" means that the isotopic enrichment is the average across the molecule (i.e. it is not a positional enrichment). For sulphur isotopes, isotopic enrichment was corrected to account for the contribution of ^{34}S: the ^{34}S-isotopologue is not labelled and comes from ^{34}S natural abundance (4.21%). It potentially complicates calculations of fluxes and sulphur allocation because unlabelled methionine and cysteine were distributed between ^{32}S and ^{34}S forms. Since sulphate used for labelling was at 98% ^{33}S and thus only contained a very small amount of ^{34}S (0.0421×0.02 = 0.84%), ^{34}S-methionine (^{34}S-cysteine) was considered to belong to the unlabeled pool and added to ^{32}S-methionine (^{32}S-cysteine).

In the case of methionine, it was possible to resolve the ^{13}C-enrichment at the different C-atom positions of the molecule thanks to LC-MS analysis with fragmentation. The isotopic enrichment of the different fragments is shown in Supplementary Figure 1. Fragments are numbered from 1 to 6 as follows (same numbering as in Supplementary Figure 1):

Fragment #	Formula	Elemental formula	Contains the CH_3 group
1	$-CH_2-S-CH_3$	C_2H_5S	Yes
2	$-CH_2-CH=CH-S-CH_3$	C_4H_7S	Yes
3	$-CH(NH_2)-CH_2-CH_2-S-CH_3$	$C_4H_{10}NS$	Yes
4	$-C(NH_2)-CH_2-CH_2-$	C_3H_6N	No
5	$-CH(NH_2)-COOH$	$C_2H_4NO_2$	No
6	$-CH_2-CH_2-CH(NH_2)-COOH$	$C_4H_8NO_2$	No

By successive mass-balance subtraction of isotopic contributions, the positional ^{13}C enrichment p at each C-atom position can be determined:

$p_{\text{overall}}(3)|p_{\text{overall}}(4) \rightarrow p(CH_3)$; or $p_{\text{overall}}(2)|p_{\text{overall}}(4) \rightarrow p(CH_3)$

$p_{\text{overall}}(6)|p_{\text{overall}}(4) \rightarrow p(COOH)$;

$p_{\text{overall}}(1)|p(CH_3) \rightarrow p(CH_2\text{-}S)$;

$p_{\text{overall}}(5)|p(COOH) \rightarrow p(CHNH_2)$;

$p_{\text{overall}}(2)|p(CHNH_2, CH_2\text{-}S, CH_3) \rightarrow p(CH_2)$

Since LC-MS data do not inform directly on the C-atom position that is ^{13}C-labelled in the signal of the $^{13}C_1$,^{33}S-isotopologue, the frequency of methionine molecules that are labelled at C-5 (methyl group) and ^{33}S simultaneously was calculated as:

$$p_{^{33}S\text{-}^{13}C5} = \frac{P-(1-L)\,p_{33}}{L}$$

Where P is the frequency of doubly labelled methionine molecules, that is, with ^{33}S and at least one ^{13}C; L is the frequency of methionine molecules that are not labelled in C-1 to C-4, calculated as $L = (1 - p_{1\text{-}4})^4$ where $p_{1\text{-}4}$ is the average ^{13}C abundance in C-1 to C-4. The absolute amount of ^{33}S,^{13}C-5-methionine was then calculated as $Q_m \cdot p_{^{33}S\text{-}^{13}C5}$ where Q_m is the amount (total pool size) of methionine (µmol m^{-2}).

Supplementary Notes 2. Calculation of the sulphate reduction rate using ^{33}S redistribution.

Assumptions and rationale. Model structure and assumptions are detailed in Supplementary Figure 2. We describe below equations used to compute the sulphate reduction rate using %^{33}S (obtained by LC-MS) and ^{33}S absolute amounts (obtained by NMR).

Here, we have neglected isotope fractionations, since they lead to negligible effects. For example, with ^{13}C, we used 99% $^{13}CO_2$ for labelling. The isotope fractionation by photosynthesis is about 20‰, meaning by definition that the quotient (atmosphere-to-fixed C) of isotope ratios (^{13}C-to-^{12}C) is $R_{atm}/R_{fixed} = 1.020$. Since CO_2 is at 99% ^{13}C, it means that $R_{atm} = 99/1 = 99$, therefore that $R_{fixed} = 99/1.020 = 97.05$. Thus, the percentage of ^{13}C in fixed carbon is $p = R_{fixed}/(1 + R_{fixed}) = 98.98\%$, which is extremely close to 99%. Therefore, the fractionation caused an extremely small modification of the isotopic signal, and could be easily neglected. The same applies to sulphur ^{33}S, if an isotope fractionation ^{32}S/^{33}S occurred in sulphate assimilation.

We calculated sulphate reduction rate using final values of both absolute ^{33}S values and %^{33}S after 2 h labelling rather than carrying out a kinetic time course of %^{33}S. There were two reasons to do so: First, it would have been impossible to sample several times the same leaf in a gas exchange system during ^{33}S labelling to carry out a kinetics analysis (for obvious reasons, the leaf could not be sacrificed several times; should multiple sacrifice have been possible, sampling itself would have perturbed CO_2/O_2 conditions when opening the chamber); Second, we avoided this problem by taking advantage of ^{33}S quantitation capabilities by NMR, so that we only required one time point per leaf. In fact, as will become apparent below, the knowledge of both ^{33}S content (U) and percentage (p) allows full resolution of the system of equations and calculation of the sulphate reduction rate.

(a) Build-up model. The differential equations associated with the change in pool size (denoted as Q) are:

Sulphate pool: $$\frac{dQ_s}{dt} = x_s F \quad (1)$$

Cysteine pool: $$\frac{dQ_c}{dt} = (1-x_s)F\cdot(1-x_c) \quad (2)$$

Methionine pool: $$\frac{dQ_m}{dt} = (1-x_s)Fx_c\cdot(1-x_m) \quad (3)$$

Differential equations describing the ^{33}S content (denoted as U) in pools are:

Sulphate ^{33}S: $$\frac{dU_s}{dt} = p_F x_s F \quad (4)$$

Cysteine ^{33}S: $$\frac{dU_c}{dt} = (1-x_s)F \cdot (p_F - x_c p_c) \quad (5)$$

Methionine ^{33}S: $$\frac{dU_m}{dt} = (1-x_s)F(x_c p_c - x_m p_m) \quad (6)$$

Where p_i stands for %^{33}S of compound i. By definition, the ^{33}S content in a given pool is the product of pool size and percentage: $U_i = Q_i \cdot p_i$. Here p_F is constant (labelling input) at 98% ^{33}S. Therefore, by substitution, equations (4-6) can be rewritten as:

Sulphate %^{33}S: $$\frac{dp_s}{dt} = \frac{x_s F}{Q_s} \cdot (p_F - p_s) \quad (7)$$

Cysteine %^{33}S: $$\frac{dp_c}{dt} = \frac{(1-x_s)F}{Q_c} \cdot (p_F - p_c) \quad (8)$$

Methionine %^{33}S: $$\frac{dp_m}{dt} = \frac{(1-x_s)x_c F}{Q_m} \cdot (p_c - p_m) \quad (9)$$

Equations (1-3) describe the total amount (regardless of S isotopes) of pools and equations (7-9) describe %^{33}S. Note that in equations (7-9), Q are not constant, i.e., pool sizes can vary with time. In fact, it has been shown that cysteine and methionine pools slowly vary with time spent in the light (*1*). p and Q can be computed numerically and x and $Q(0)$ values can be solved so that both modelled p and U values at $t = 7200$ s match the final values observed (total time of labelling pulse). $Q_s(t = 0)$ is known since the ^{33}S signal of natural leaf sulphate (without labelling) was measured by NMR (denoted as U_s^0) and thus $Q_s(t = 0) = U_s^0/p_o$ where p_o is ^{33}S natural abundance (0.76%). $p_c(t = 0)$ and $p_m(t = 0)$ are set at ^{33}S natural abundance p_o. The sulphate reduction rate is $(1-x_s)F$.

(b) Recycling model. Recycling could not be incorporated in the build-up model otherwise the system of equations would have been underdetermined. The build-up model (where pool sizes are not constant) and the recycling model (were pools are constant but there is a possible isotopic dilution caused by metabolic recycling) are thus two extreme cases useful to examine the robustness of calculated sulphate reduction rate with respect to modelling assumptions: in model (a), ^{33}S-cysteine is turned-over by its consumption; in model (b), ^{33}S-cysteine is isotopically diluted by the recycling of cysteine at natural abundance. Since the model assumes a steady-state of cysteine and methionine pool sizes, we have:

$$\frac{dQ_c}{dt} = \frac{dQ_m}{dt} = 0 \quad (10)$$

The pool of sulphate is not in the steady-state and there is a build-up of ^{33}S-sulphate so that equation (1) remains valid. Equation (10) implies that:

$$\frac{dU_c}{dt} = Q_c \frac{dp_c}{dt} \text{ and } \frac{dU_m}{dt} = Q_m \frac{dp_m}{dt} \quad (11)$$

Therefore, by mass-balance, we have:

Cysteine %^{33}S: $$\frac{dp_c}{dt}=\frac{(1-x_s)F(p_F-p_c)+r_c(p_o-p_c)}{Q_c} \quad (12)$$

Methionine %^{33}S: $$\frac{dp_m}{dt}=\frac{((1-x_s)F+r_c)(p_c-p_m)+r_m(p_o-p_m)}{Q_m} \quad (13)$$

Equations (12-13) describe the %^{33}S in cysteine and methionine and can be computed numerically to solve $(1–x_s)F$, r_m, r_c so that modelled p and U values at t = 7200 s match observed final values (total time of labelling pulse). As before, $Q_s(t = 0)$ is known since the ^{33}S signal of natural leaf sulphate (without labelling) is known. $p_c(t = 0)$ and $p_m(t = 0)$ are set at ^{33}S natural abundance p_o. Resolution using model (a) or (b) is illustrated in Supplementary Figure 6.

Supplementary Notes 3. Calculation of ^{13}C utilization and sulphate reduction rate with ^{13}C

The metabolic framework used here for calculations is shown in Supplementary Figure 7.

%^{13}C in metabolically active glycine. To estimate the %^{13}C in metabolically active serine and glycine, we use the assumption that isotopic redistribution in Calvin cycle intermediates is rapid therefore there is an equal probability to label C-atom positions in RuBP. This assumption is justified because labelling time is long (2 h) and therefore, the chloroplastic pool of RuBP is probably totally ^{13}C-labelled. Thus, the probability of finding two ^{13}C atoms in metabolically active glycine is the square of the probability of having labelling at a given position. That is, quantities of isotopomers should be as follows:

^{12}C-glycine: $$^{12}U=(1-p_o)^2 Q_{\text{inact}}+(1-p_g)^2 Q_{\text{act}} \quad (14)$$

^{13}C$_1$-glycine (either in C-1 or C-2): $$^{13}U_1=2p_o(1-p_o)Q_{\text{inact}}+2p_g(1-p_g)Q_{\text{act}} \quad (15)$$

^{13}C$_2$-glycine: $$^{13}U_2=p_o^2 Q_{\text{inact}}+p_g^2 Q_{\text{act}} \quad (16)$$

Where Q_{inact} and Q_{act} are pool sizes of static and active glycine pools, p_o is natural abundance (1.1%) and p_g is the %^{13}C of metabolically active glycine. Clearly, because natural abundance is only 1.1%, p_o^2 is very small and ^{13}C$_2$-glycine is representative of metabolically active glycine. Therefore:

$$^{12}U\approx(1-a)Q_{\text{inact}}+(1-p_g)^2 Q_{\text{act}} \quad (17)$$

$$^{13}U_1\approx aQ_{\text{inact}}+2p_g(1-p_g)Q_{\text{act}} \quad (18)$$

$$^{13}U_2\approx p_g^2 Q_{\text{act}} \quad (19)$$

Where $a = 2p_o = 0.022$. Note that U values can be normalized using $Q_{\text{act}} + Q_{\text{inact}} = {}^{12}U + {}^{13}U_1 + {}^{13}U_2 = Q_{\text{total}}$ showing that units do not have any impact on calculations that follow. Combining (14) and (15), we have:

$$^{13}U_1-{}^{12}U\frac{a}{1-a}=\left(2p_g(1-p_g)-\frac{a}{1-a}(1-p_g)^2\right)\cdot Q_{\text{act}} \quad (20)$$

With (19), (20) gives (note that Q_{act} disappears):

$$\frac{{}^{13}U_2}{{}^{13}U_1 - {}^{12}U\dfrac{a}{1-a}} = \frac{{p_g}^2}{2p_g\left(1-p_g\right) - \dfrac{a}{1-a}\left(1-p_g\right)^2} \quad (21)$$

Using observed values of U (using LC-MS), equation (21) defines a relationship of degree 2 that can be solved for p_g. Then $Q_{\text{act}}/Q_{\text{total}}$ can be calculated using equation (19).

%[13]C in metabolically active serine. Assuming that the %[13]C in source glycine is p_g (calculated above), serine isotopomer abundance is as follows:

^{12}C-serine: $${}^{12}U = \left(1-a\right)Q_{\text{inact}} + (1-p_g)^2\left(1-p_p\right)Q_{\text{act}} \quad (22)$$

$^{13}C_1$-serine: $${}^{13}U_1 = aQ_{\text{inact}} + \left(2p_g\left(1-p_g\right)\left(1-p_p\right) + p_p\left(1-{p_g}^2\right)\right)Q_{\text{act}} \quad (23)$$

$^{13}C_2$-serine: $${}^{13}U_2 = \left({p_g}^2\left(1-p_p\right) + 2p_p p_g\left(1-p_g\right)\right)Q_{\text{act}} \quad (24)$$

$^{13}C_3$-serine: $${}^{13}U_3 = {p_g}^2 p_p Q_{\text{act}} \quad (25)$$

Where p_p is the %[13]C in mitochondrial C_1-H_4F pool (which is at the origin of the C-3 atom of serine). A simplistic assumption would be to set p_p equal to p_g since the hydroxymethyl group comes from decarboxylated glycine (while two C-atoms in serine come directly from glycine). However, there could be an isotopic difference between p_g and p_p due to the rapid isotopic dilution in the mitochondrial C_1-H_4F pool. Calculating the ${}^{13}U_3$-to-${}^{13}U_2$ ratio eliminates Q_{act} and thus allows the calculation of p_p:

$$p_p = \frac{1}{3 + \dfrac{{}^{13}U_2}{{}^{13}U_3} - \dfrac{2}{p_g}} \quad (26)$$

Using equation (25), the knowledge of p_p and p_g allows the calculation of Q_{act} (or similarly, $Q_{\text{act}}/Q_{\text{total}}$) and using equation (22), we may calculate Q_{inact} with $a = 3p_o = 0.033$.

Cysteine de novo synthesis. Cysteine derives from serine and thus the proportion of cysteine molecules that inherit C-atoms of photorespiratory serine (denoted as x_{photo}) is:

$$x_{\text{photo}} = \frac{p_{cys} - p_o}{p_{ser} - p_o} \quad (27)$$

Where p_{cys} and p_{ser} are overall %[13]C in cysteine and serine (but note that this calculation can be done on a positional basis). The apparent flux of de novo cysteine synthesis from metabolically active serine is:

$$s_{c\,\text{app}} = \frac{x_{\text{photo}} \cdot Q_c}{T} \quad (28)$$

Where Q_c is cysteine pool size. However, equation (28) provides an average value that does not account for the dynamics of isotopic labelling in serine (and potentially, of pool size). The flux of de novo cysteine synthesis, should verify:

$$\frac{dp_{cys}}{dt} = \frac{s_c}{Q_c}\left(p_{ser} - p_{cys}\right) \quad (29)$$

Note that in (29), p_{ser} vary with time (as well as Q_c, see SM5). If the total serine pool is assumed to be constant, one may describe the time course of p_{ser} as:

$$\frac{dp_{ser}}{dt} = \frac{v_o}{2Q_{ser\ act}}\left(p_g - p_{ser}\right) \tag{30}$$

Where v_o is the oxygenation rate (estimated from gas exchange), and p_g determined as above. $Q_{ser\ act}$ can be calculated using the ratio Q_{act}/Q_{total} (see above) and the absolute amount of serine measured by LC-MS. (29) can then be solved numerically to calculate s_c (with $p_{cys}(t = 0) = 0.011$).

Supplementary references

1. C. Abadie, S. Blanchet, A. Carroll, G. Tcherkez, Metabolomics analysis of postphotosynthetic effects of gaseous O_2 on primary metabolism in illuminated leaves. *Functional Plant Biology* **44**, 929-940 (2017).
