# Supplementary material for: Plant sulphur metabolism is stimulated by photorespiration
Source: Commun Biol. 2019 Oct 16;2:379. doi: 10.1038/s42003-019-0616-y (PMC6795801; doi:10.1038/s42003-019-0616-y)
Supplement: Supplementary file 2 — Description of Additional Supplementary Files [file 42003_2019_616_MOESM2_ESM.pdf]

Description of additional supplementary items

Source data associated with plots presented in main figures are available as Supplementary Data 1 (Excel file): Source Data Fig. 2 (isotopic enrichment), and Source Data Fig. 3 (S-flux), as two separate sheets. When applicable, units associated with numbers are recalled on top of Excel sheets.
